# Automatic detection of standing dead trees based on improved YOLOv7 from airborne remote sensing imagery

**DOI:** 10.3389/fpls.2024.1278161

**Published:** 2024-01-22

**Authors:** Hongwei Zhou, Shangxin Wu, Zihan Xu, Hong Sun

**Affiliations:** ^1^ College of Computer and Control Engineering, Northeast Forestry University, Harbin, China; ^2^ Key Laboratory of National Forestry and Grassland Administration on Forest and Grassland Pest Monitoring and Warning, Center for Biological Disaster Prevention and Control, National Forestry and Grassland Administration, Shenyang, China

**Keywords:** standing dead trees, deep learning, attention mechanism, Wise-IoU loss function, airborne remote sensing imagery

## Abstract

Detecting and localizing standing dead trees (SDTs) is crucial for effective forest management and conservation. Due to challenges posed by mountainous terrain and road conditions, conducting a swift and comprehensive survey of SDTs through traditional manual inventory methods is considerably difficult. In recent years, advancements in deep learning and remote sensing technology have facilitated real-time and efficient detection of dead trees. Nevertheless, challenges persist in identifying individual dead trees in airborne remote sensing images, attributed to factors such as small target size, mutual occlusion and complex backgrounds. These aspects collectively contribute to the increased difficulty of detecting dead trees at a single-tree scale. To address this issue, the paper introduces an improved You Only Look Once version 7 (YOLOv7) model that incorporates the Simple Parameter-Free Attention Module (SimAM), an unparameterized attention mechanism. This improvement aims to enhance the network’s feature extraction capabilities and increase the model’s sensitivity to small target dead trees. To validate the superiority of SimAM_YOLOv7, we compared it with four widely adopted attention mechanisms. Additionally, a method to enhance model robustness is presented, involving the replacement of the Complete Intersection over Union (CIoU) loss in the original YOLOv7 model with the Wise-IoU (WIoU) loss function. Following these, we evaluated detection accuracy using a self-developed dataset of SDTs in forests. The results indicate that the improved YOLOv7 model can effectively identify dead trees in airborne remote sensing images, achieving precision, recall and mAP@0.5 values of 94.31%, 93.13% and 98.03%, respectively. These values are 3.67%, 2.28% and 1.56% higher than those of the original YOLOv7 model. This improvement model provides a convenient solution for forest management.

## 1 Introduction

Trees are essential for maintaining the ecological balance within forest ecosystems (Manning et al., 2006; Nadrowski et al., 2010). Diseases and pests are significant factors contributing to the widespread death of trees (Bernal et al., 2023; Luo et al., 2023; Wang J. et al., 2023). Regularly inspecting standing dead trees (SDTs) in the forest to determine the causes of their death facilitates early detection and the mitigation of potential pest and disease issues. Therefore, it is essential to accurately and efficiently identify and monitor dead trees in forest areas. Traditional SDTs inventory methods often rely on rangers collecting coordinate location information in the field. However, this approach is hindered by challenging mountainous terrain and road conditions. Field trekking for inventory purposes becomes difficult, costly, and time-consuming (Butler and Schlaepfer, 2004).

To complement field trekking, low- and medium-resolution satellite remote sensing images have been used to detect the extent of forest infestation in localized areas (Eklundh et al., 2009; Coops et al., 2010; Meng et al., 2016). However, these studies have primarily focused on area-based detection, lacking the ability to identify disease-infected dead trees at the single-tree scale. With advancements in remote sensing platforms and technologies, the use of high-resolution satellite remote sensing images (e.g., QuickBird, IKONOS) and aerial images has made it possible to detect single tree. By combining these images with canopy detection methods, more accurate identification of dead trees, even in mountainous areas with challenging terrain and rugged roads, has become achievable (Hicke and Logan, 2009; Wang et al., 2015; Windrim et al., 2020). While Light Detection and Ranging (LiDAR) technology can provide precise information on the location and height of individual SDTS in forests, it comes with a high cost for data collection (Chen et al., 2011). On the other hand, high-resolution optical remote sensing images offer several advantages, such as easy data collection and wide application, making them a prominent focus for research on single-tree identification techniques (Han et al., 2022; Lee et al., 2023; Zheng et al., 2023).

With the proposal and development of machine learning methods, they have been utilized by scholars to detect dead trees using high-resolution remote sensing images (Maxwell et al., 2018). These methods involve the application of various machine learning algorithms such as Support Vector Machine (SVM), Random Forest (RF), k-Nearest Neighbor Algorithm (K-NN), Clustering Algorithm and Artificial Neural Network (ANN) for SDTs detection (Celik, 2009; Lee et al., 2019; Miltiadou et al., 2020). However, the existing methods often rely on manual design and extraction of image features, which can limit their accuracy and robustness, particularly in complex scenarios such as tree occlusion or when dealing with similar colors of features. Accurately detecting dead trees using machine learning methods becomes challenging due to these limitations.

In recent years, deep learning has made significant advancements, leading to the development of powerful object detection models based on convolutional neural net-works (CNN). Compared to traditional machine learning methods, deep learning models have the ability to automatically learn image features during the detection of SDTs (Farias et al., 2018). They can also synthesize contextual information and semantic relationships within the image, enhancing detection accuracy (Lei et al., 2021; Zhang et al., 2022). The end-to-end training approach simplifies the dead trees detection system and has the potential to improve overall performance and efficiency. Deep learning algorithms based on CNN have demonstrated advantages over other methods, leading researchers across various fields to explore their application (Voulodimos et al., 2018; Naga Srinivasu et al., 2023). In the field of forestry, deep learning has been widely used for tasks such as forest resource management, soil analysis, tree detection and classification (Srivastava et al., 2021; Wang et al., 2021). Scholars have conducted research on dead tree detection based on deep learning (Chiang et al., 2020; Li et al., 2022; Wang et al., 2022). They primarily focus on detecting larger or densely packed targets, with limited studies addressing the detection of individual dead trees at a smaller scale. However, there are several challenges in achieving SDTs detection at the single-tree scale: 1) Multi-scale problem: SDTs exhibit variations in size dimensions and shapes, making accurate localization challenging. The detection models need to account for these multi-scale variations to accurately identify dead trees. 2) Occlusion problem: In remote sensing images, the presence of living trees can obscure SDTs, making it difficult to distinguish their boundaries and features, which could lead to missed detections. 3) Background complexity: Remote sensing images may contain complex backgrounds, including houses, land, bare rocks or other elements. This complexity can result in misidentifications, where the background is mistakenly detected as SDTs.

Deep learning-based object detection algorithms can be broadly categorized into two types based on the presence of a candidate region extraction step: two-stage algorithms, exemplified by Faster R-CNN, and single-stage algorithms, represented by You Only Look Once (YOLO). While two-stage algorithms, like Faster R-CNN, typically exhibit slower detection speeds due to their two-step nature, they often achieve higher detection accuracy. On the other hand, YOLO, with its continuous improvements in network architecture, demonstrates advanced performance in both detection accuracy and speed (Sirisha et al., 2023). This paper presents an improved algorithm based on the You Only Look Once version 7 (YOLOv7) model to address the challenges of detecting small targets, mutual occlusion and complex backgrounds in optical remote sensing images for automated SDTs detection. The contributions of this paper can be summarized as follows:

1. Introducing the Simple Parameter-Free Attention Module (SimAM) to enhance the model’s feature extraction capabilities for small target dead standing trees.
2. Replacing the Complete Intersection over Union (CIoU) loss with Wise-IoU (WIoU) improves the robustness and detection accuracy of the model by focusing on ordinary quality bounding box.
3. Analyzing the performance metrics of the proposed improved model, including precision, recall, mAP@0.5 and Frames Per Second (FPS), against a benchmark model.
4. Discussing the research advancements in SDTs detection using deep learning methods, along with the limitations of this study and future research directions.

The paper is structured as follows: the introduction offers the research area, outlines the authors’ contributions and elucidates the workflow of the model detection. Section 2 details the experimental materials and introduces the proposed model for SDTs detection. Section 3 showcases the experimental results. Discussion and conclusions are presented in Section 4 and Section 5.

**Figure 1:**
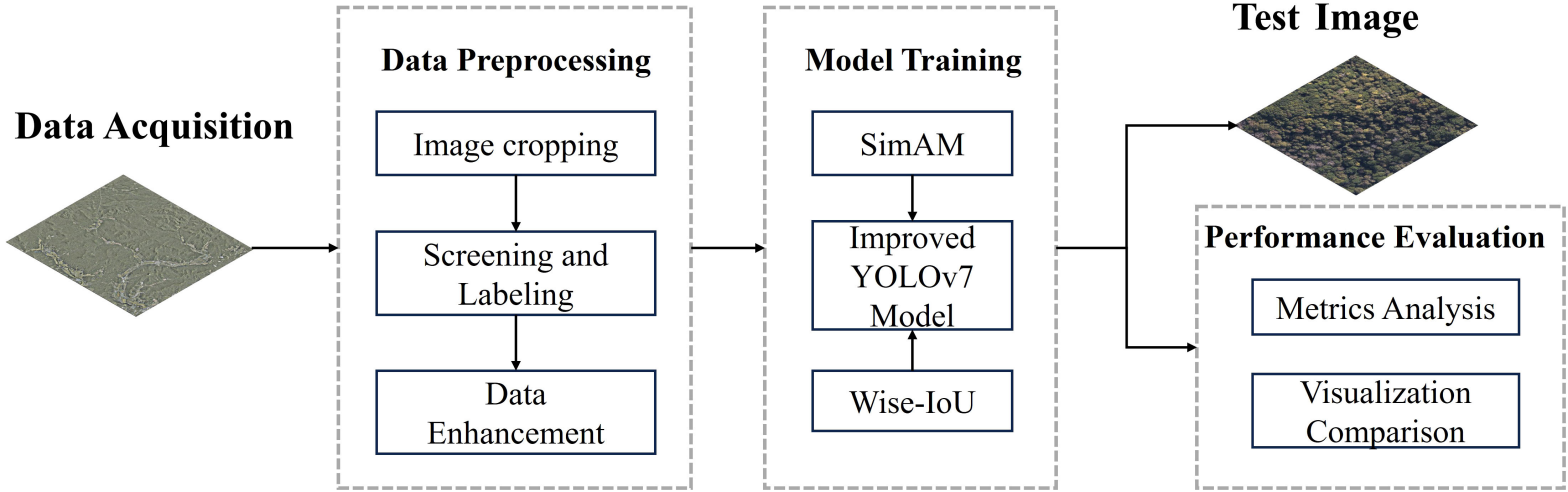
The workflow diagram of SDTs detection model.

The workflow diagram of SDTs detection model based on the improved YOLOv7 algorithm is shown in **Figure 1**. First, the collected remote sensing images are preprocessed, including three parts: image cropping, screening and labeling, and data enhancement. Then the images are fed into the improved YOLOv7 network for training to obtain the training model. The SDTs in the test set of images are detected with the training model, and finally the model detection effect is comprehensively evaluated by combining various evaluation indexes and visualization results.

## 2 Materials and methods

### 2.1 Study areas and dataset

**Figure 2:**
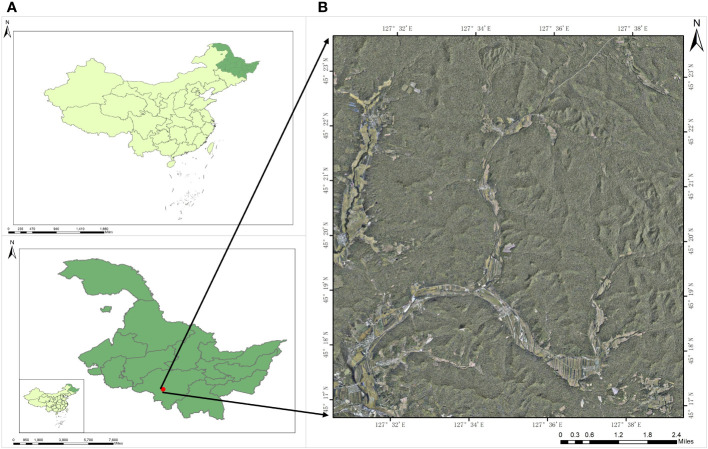
Location of the study area; **(A)** Map of Heilongjiang Province, China, with red dots representing the study area. **(B)** Geographic location of the study area.

The study area for this experiment is the Experimental Forestry Farm of Mao’er Mountain, located in the southeastern part of Heilongjiang Province, China. It is situated in the northwestern part of Shangzhi City, on the western slope of Zhang Guangcailing, within the geographic coordinates of 45°16′ to 45°24′N and 127°30′ to 127°40′E (**Figure 2**). The Forestry Farm is divided into ten sizing zones, characterized by numerous mountainous hills with gentle slopes and elevations ranging from 200 to 600 meters above sea level. The climate in Mao’er Mountain belongs to the temperate continental monsoon climate. It experiences long and dry winters, short and warm summers, concentrated rainfall, frequent spring droughts, and occasional fall freezes. The area has an annual frost-free period of approximately 125 days and an average annual precipitation of around 700mm. Being the source of the Ash River and the Ujimi River, the area is fertile, and the soil primarily consists of dark brown loam. The soil is rich in trace elements and organic matter, accounting for approximately 68.18% of the forest area. Since its establishment, the Mao’er Mountain Experimental Forestry Farm has developed significant forestry land, covering an area of 26,000 hectares, with a forest coverage rate of 83.29%. It possesses abundant forest resources, playing a crucial role in maintaining the ecological balance of the region.

Due to the limited scope of existing research on SDTs and the absence of publicly available datasets, we conducted this study using a self-built dataset. In this study, the study area was photographed and scanned using a high-resolution CCD sensor (DigiCAM-60) carried by the LiCHy system to obtain 27.4G of raw remote sensing image data. The flight altitude was 1000m and the day of shooting was clear and cloudless. **Table 1** shows the detailed parameters of the CCD sensor.

**Table 1:** Main parameters of CCD sensors.

Main parameters	Parameter value	Main parameters	Parameter value
Frame Size	8956*6708	Pixel Size	0.25m*0.25m
Imaging Sensor Size	3ns	Bit Depth	16bits
FOV	56.2°	Focal Length	50mm
Ground Resolution	0.12m		

To prepare the original RGB images from the CCD sensor for target recognition, a series of preprocessing steps are performed. First, considering that the mismatch of aspect ratio may affect the training effect of the model, the original image is cropped to 10824 RGB images of 3*1024*1024 uniform size using ArcGIS software.

**Figure 3:**
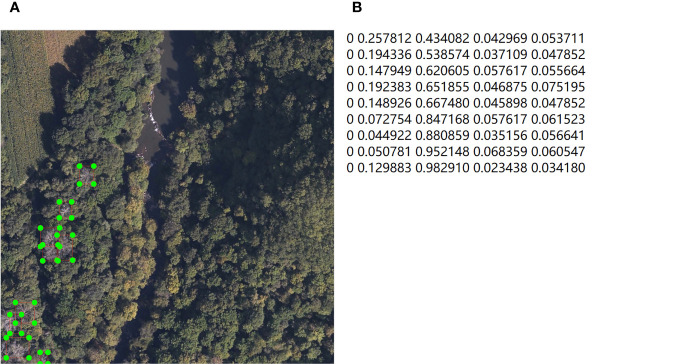
Data annotation; **(A)** label of SDTs; **(B)** annotations files of image.

Secondly, not every image in the dataset contains SDTs, and images with dead trees have significantly fewer instances compared to healthy trees. Considering the balance of samples in the object detection task and the limited computational resources, in order to allow the model to focus more on the core objective, which is the detection of dead trees, we need to filter out images containing SDTs. From the entire dataset, 1928 im-ages containing dead trees are selected. Using the labelimg software, we annotated dead trees in the images to create labels in VOC format for subsequent comparative experiments. These VOC format labels were then converted into YOLO format. The study primarily focused on detecting dead tree crowns using RGB images. During annotation, the focus was on distinguishing dead trees from the background, requiring marking only the crowns of dead trees. By combining visual interpretation and on-site surveys, specific rules were established: the crown of an individual tree was the target, and if all its tree tops showed signs of death, it was labeled as a dead tree (category “0”). The labeling result is shown in the **Figure 3**, the annotation file of image is used to represent the label category and the coordinates of the rectangular marking box.

**Figure 4:**
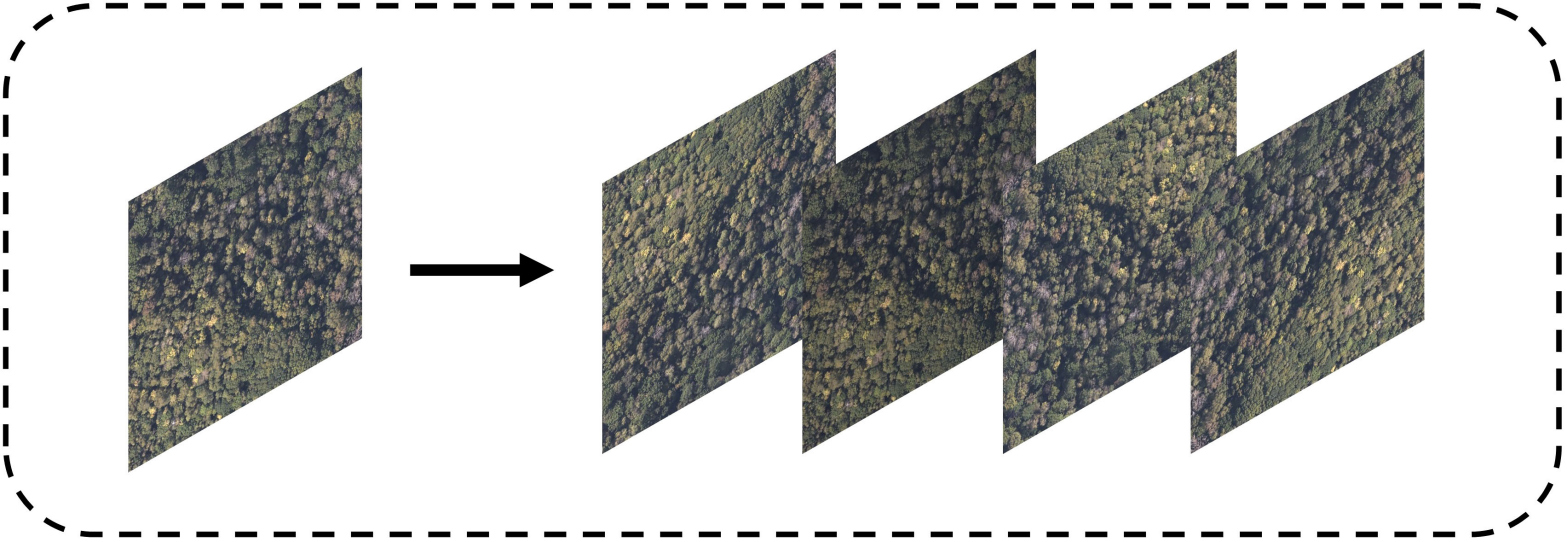
Some samples after data enhancement.

To enhance the model’s robustness and generalization ability while avoiding overfitting, data augmentation techniques are applied. The dataset is expanded through random flipping, mirroring, and luminance adjustments. This augmentation process generates a total of 9640 dataset samples. **Figure 4** showcases some of the samples after data enhancement. The labeled dataset is then di-vided into training, validation, and test sets in a ratio of 6:2:2. This division results in a total of 5784 training set samples, 1928 validation set samples, and 1,928 test set samples for this experiment.

### 2.2 Related work

#### 2.2.1 YOLOv7

This paper presents an algorithm based on the YOLOv7 for detecting dead standing trees in large forests. The algorithm aims to enable rangers to accurately locate dead standing trees quickly, which is crucial for the maintenance of forest resources and biodiversity. Given the requirements for accuracy and real-time performance in SDTs detection, the YOLOv7 model is chosen as the foundation for detecting and locating dead trees.

**Figure 5:**
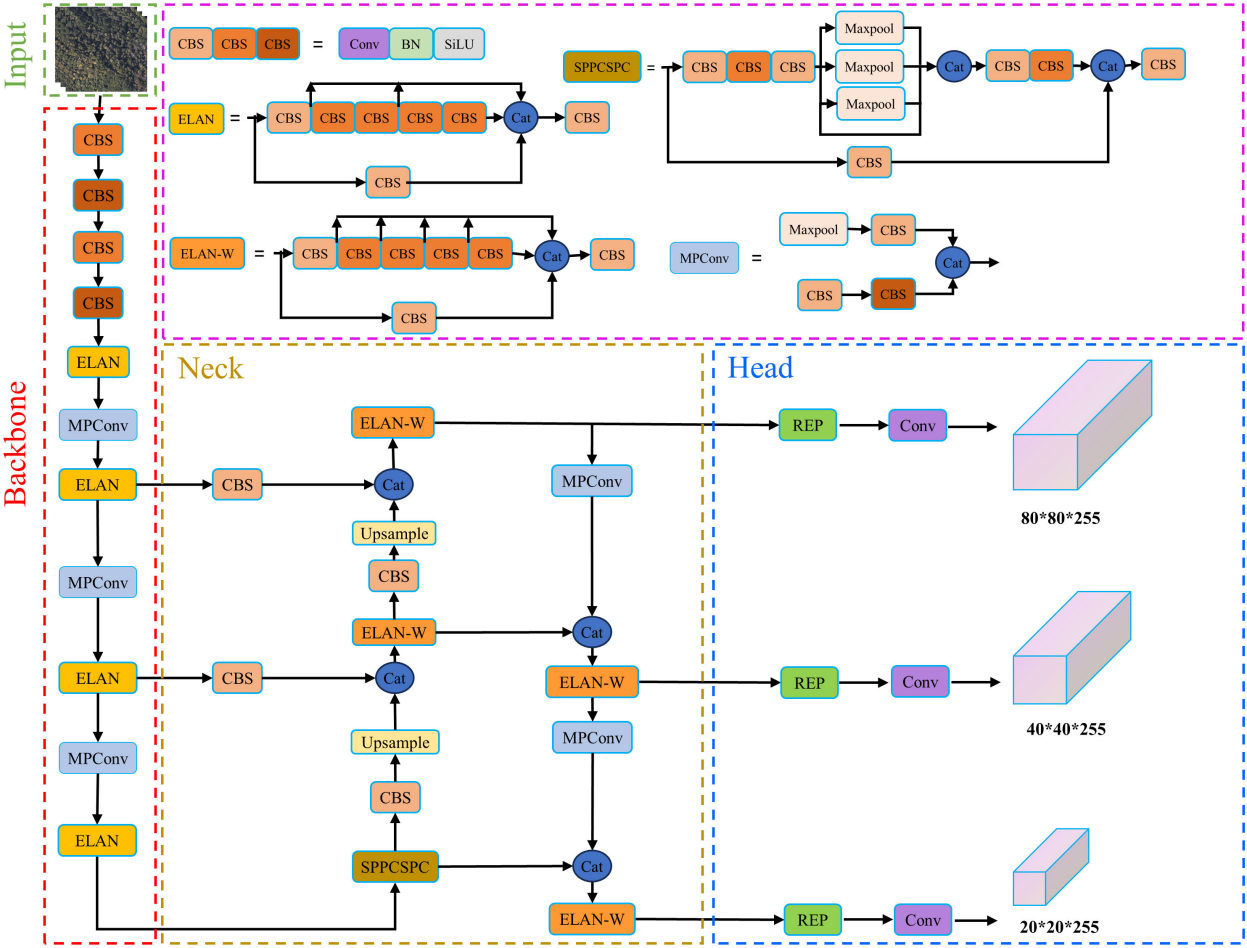
Structure of YOLOv7 network.

The YOLOv7 (Wang C. et al., 2023) comprises three basic models with increasing parameter counts: YOLOv7-tiny for edge GPU, YOLOv7 for normal GPU, and YOLOv7-w6 for cloud GPU. Additionally, there are four extended models based on the basic models, namely YOLOv7-X, YOLOv7-E6, YOLOv7-D6, and YOLOv7-E6E. The model structure of YOLOv7 is illustrated in **Figure 5**. The overall detection logic of YOLOv7 is similar to that of YOLOv4 and YOLOv5.

The YOLOv7 model consists of four main components: input, backbone, neck and head. The input component performs preprocessing operations such as online data enhancement and resizing on the original image to obtain a 640*640*3 RGB image. In **Figure 5**, we use the abbreviation ‘Cat’ to represent ‘concatenate,’ which is employed to concatenate the outputs of multiple feature maps or branches along the channel axis. This is done to provide a richer feature representation and is commonly used for multi-scale information fusion. The backbone component is responsible for feature extraction from the input RGB image. In the YOLOv7 model, the backbone utilizes the ELAN module, which controls the shortest and longest gradient paths to achieve more effective learning and convergence. It generates three feature maps that serve as inputs to the neck.

The neck component is responsible for multi-scale feature fusion. It introduces the SPPCSPC module and optimizes the PAN module. The SPPCSPC module combines the Spatial Pyramid Pooling (SPP) structure with the Cross Stage Partial (CSP) structure. The role of the SPP structure is to obtain different receptive fields through max-pooling, and the role of the CSP structure is to divide the input feature map into two parts. Each part is separately processed through a subnetwork and in the subsequent layer. The two parts of the feature map are then concatenated as the input for the next layer. The SPPCSPC module combines the advantages of both structures by parallel processing the features into two parts. Only one part undergoes SPP structure processing, and the final step involves concatenating the two parts to reduce model computation and improve training speed. The PAN module further enhances the model’s learning capability by introducing an ELAN-W structure, which is similar to that used in the backbone. This structure improves learning without changing the gradient paths. The PAN module efficiently fuses multi-scale feature maps, enabling the model to learn and capture information at different scales effectively.

The head component is responsible for predicting image features. It incorporates the RepConv design, which utilizes a heavily parameterized convolutional architecture to enrich the gradient diversity of feature maps at different scales. This reduces model complexity, enhances the model’s prediction ability, and predicts the bounding box location and confidence information of SDTs using three feature maps.

#### 2.2.2 Attention mechanisms

When performing a visual task, human vision will quickly focus towards important regions and prioritize limited attention to process the critical part of the task, researchers propose to process data more efficiently by incorporating an attention mechanism based on this characteristic of human vision. In recent years, the attention mechanism, as a plug-and-play and very effective module, has been widely used in a variety of deep learning tasks such as natural language processing, computer vision and data prediction (Niu et al., 2021).

The combination of attention mechanism and convolutional neural network is the focus of research in the field of computer vision, and the addition of the attention mechanism enables the model to focus its attention on the object region of the image (Kim and Verghese, 2012), differentiating from processing the whole image, focusing on extracting the object region features, and effectively improving the model performance. In terms of the object detection task in the field of computer vision, the introduction of the attention mechanism can make the object feature extraction more adequate, reduce the interference of the background image and negative samples (Chai et al., 2023), and realize the effective improvement of the model detection performance.

In this paper, several experiments are conducted with the YOLOv7 model, and it is found that the model is not suitable for feature extraction of small targets in remote sensing images, which exhibits issues of leakage and misdetection when detecting some SDTs. Therefore, the attention module is added to the YOLOv7 model to improve its characterization ability and further improve the model detection accuracy.

#### 2.2.3 Bounding box regression loss function

Traditional target localization usually uses the Mean Square Error (MSE) loss function to compute the coordinates of the predicted bounding box centroid as well as the loss of width and height (Redmon et al., 2016), which directly estimates the offset and is susceptible to the interference of outliers and poor robustness. To address the limitations of traditional BBR methods, researchers have proposed several improved loss functions. Ross Girshick introduced the Intersection over Union (IoU) loss (Girshick, 2015), which calculates the intersection and concurrency ratio between the predicted and true bounding boxes. This loss function reduces the impact of large-scale bounding boxes on the model’s loss. However, it lacks attention to the non-overlapping area between the two bounding boxes. To overcome this, the Generalized-IoU (GIoU) loss (Rezatofighi et al., 2019) was proposed, which uses the area of the smallest bounding box that encloses both boxes as the denominator, providing a better measure of overlap. The Distance-IoU (DIoU) loss incorporates the distance between the centroids of the two bounding boxes into the loss function, further improving the detection performance. Building upon the DIoU loss, the Complete-IoU (CIoU) loss incorporates the aspect ratio into the calculation of the loss function (Zheng et al., 2022). This enhancement improves the convergence speed of model training and the accuracy of bounding box detection.

In the YOLOv7 model, the CIoU loss is used for BBR. However, it does not fully consider the balance between high and low-quality examples. To reduce the impact of low-quality example regression on the detection performance, this paper adopts a more balanced gradient allocation method. By focusing the loss function mainly on ordinary quality bounding boxes, the detection performance of the model is further improved.

### 2.3 Improved YOLOv7 SDTs detection model

#### 2.3.1 SimAM attention mechanism

SDTs detection is challenging due to the complexity and variability of target scale and picture background. Remote sensing images often contain irrelevant features like roads and houses, which can interfere with dead trees detection. Moreover, the distribution of dead trees in the images can be diverse, requiring high-performance detection and localization by the model.

Taking inspiration from Li, Y (Li et al., 2023), who proposed the Attention-YOLOv4 algorithm to reduce background interference in detecting small target traffic signs, this paper proposes the introduction of the SimAM module (Yang et al., 2021) to improve the model’s anti-interference ability in dead trees detection. The SimAM module, based on visual neuro-science theory, optimizes the design of the energy function to compute different neuron weights. It provides a fast closed-form solution for the optimized energy function, enhancing the feature extraction capability of the model without introducing additional parameters or increasing computational complexity.

The minimum energy function effectively reduces the computational amount while calculating the corresponding weights of each neuron in the dead trees feature map and discriminates the linear differentiability among neurons, the minimum energy function can be expressed by Equations 1–3:


(1) $$e_{t}^{*}=\frac{4\left({\hat{\sigma }}^{2}+\lambda \right)}{{\left(t-\hat{\mu }\right)}^{2}+2{\hat{\sigma }}^{2}+2\lambda }$$

(2) $$\hat{\mu }=\frac{1}{M}\sum_{i=1}^{M}x_{i}$$

(3) $${\hat{\sigma }}^{2}=\frac{1}{M}\sum_{i=1}^{M}{\left(x_{i}-\hat{\mu }\right)}^{2}$$

In the equation, t represents the target neuron in a single channel of the feature map, x represents the other neurons, M represents the number of neurons in a single channel, λ represents the canonical term, and $\hat{\mu }$ and ${\hat{\sigma }}^{2}$ represent the mean and variance of the other neurons in a single channel, respectively. According to the above equations, it can be seen that the smaller the minimum capability $e_{t}^{*}$ is, the more linearly separable the target neuron is from other neurons in a single channel, and the more critical it is for model feature extraction. The weights corresponding to each neuron in the feature map can be obtained from $1/e_{t}^{*}$ . Finally, the model undergoes overall refinement through the scaling operator (Equation 4).


(4) $$\tilde{X}=\text{sigmoid}\left(\frac{1}{E}\right)☉X$$

**Figure 6:**
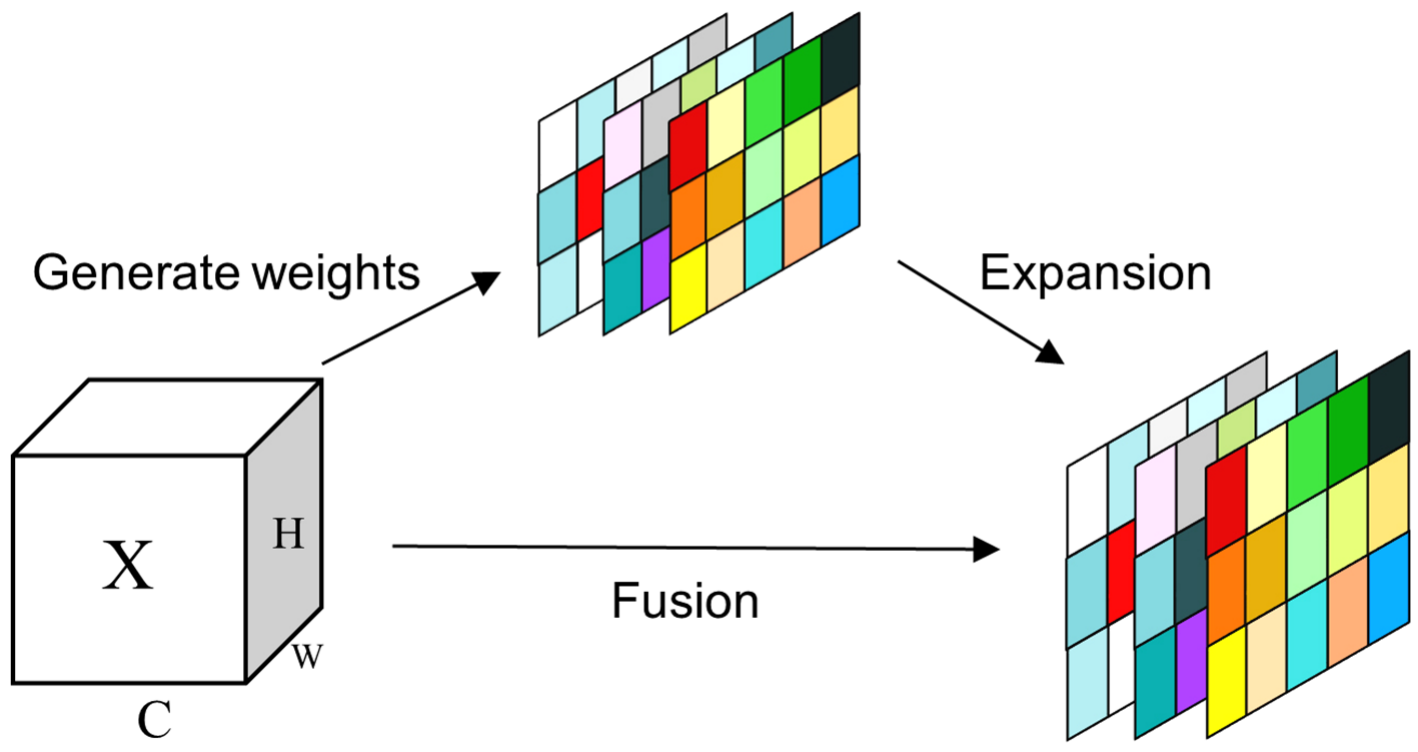
SimAM attention mechanism.

Where E groups the minimum energy of all neurons, Sigmoid function is used to prevent the value of E from being too large. The structure of SimAM is shown in **Figure 6**. The feature map is fed into the SimAM attention mechanism to get the weights of each neuron and then normalized. Then each neuron of the original feature map is multiplied by the corresponding weights to obtain the output feature map.

#### 2.3.2 Improved YOLOv7 model with introduction of SimAM attention mechanism

**Figure 7:**
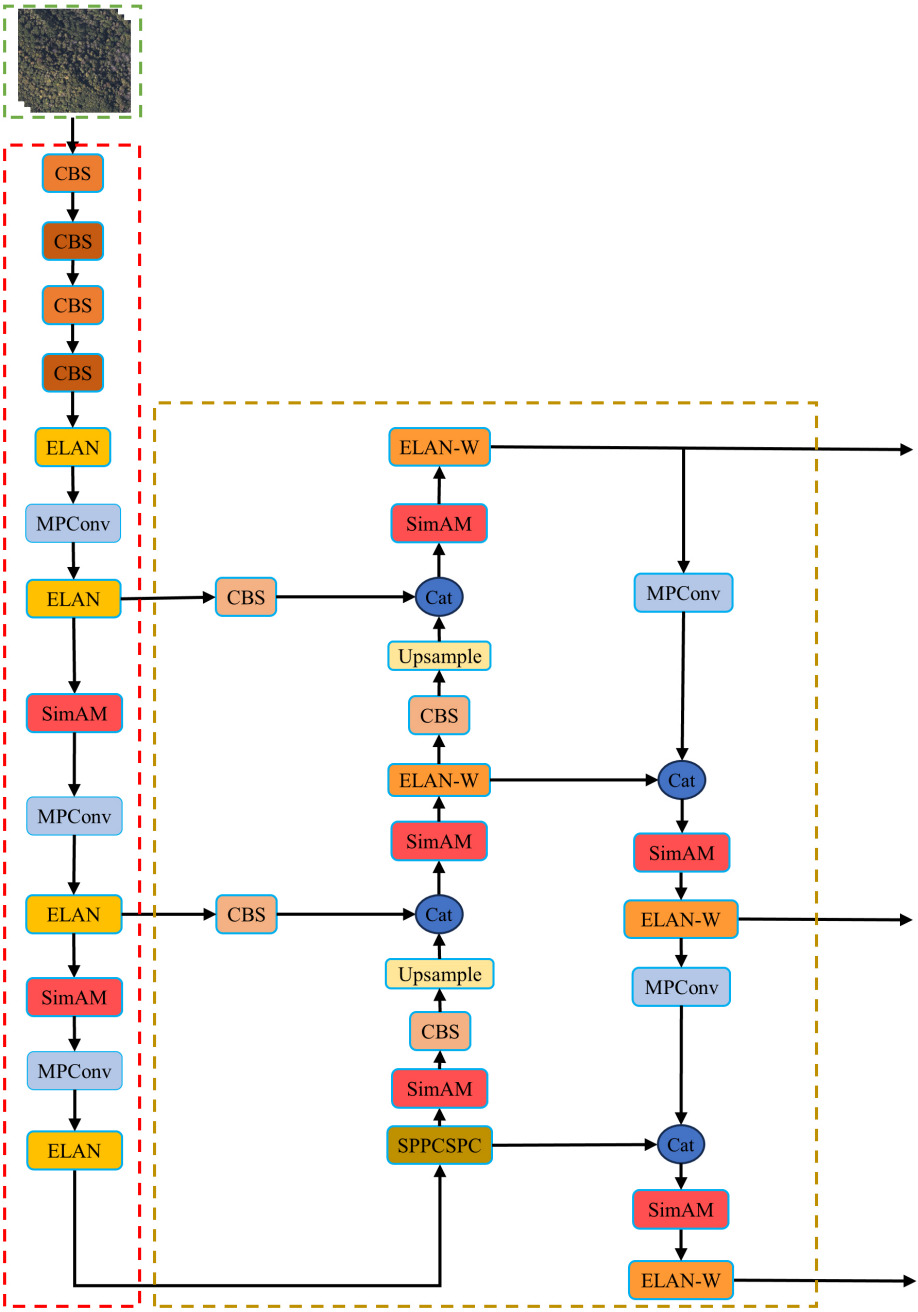
SimAM module embedded design.

SimAM is a plug-and-play module that enhances the network’s representational ability by computing 3D weights, unlike channel and spatial attention mechanisms that treat each neuron equally. In the YOLOv7 network, the SimAM attention mechanism is incorporated into backbone and neck feature extraction network. This module aims to focus more on the detailed features of SDTs and improve the model’s detection performance. The structure of the YOLOv7 network with the SimAM attention mechanism is depicted in **Figure 7**.

#### 2.3.3 Wise-IoU loss function

**Figure 8:**
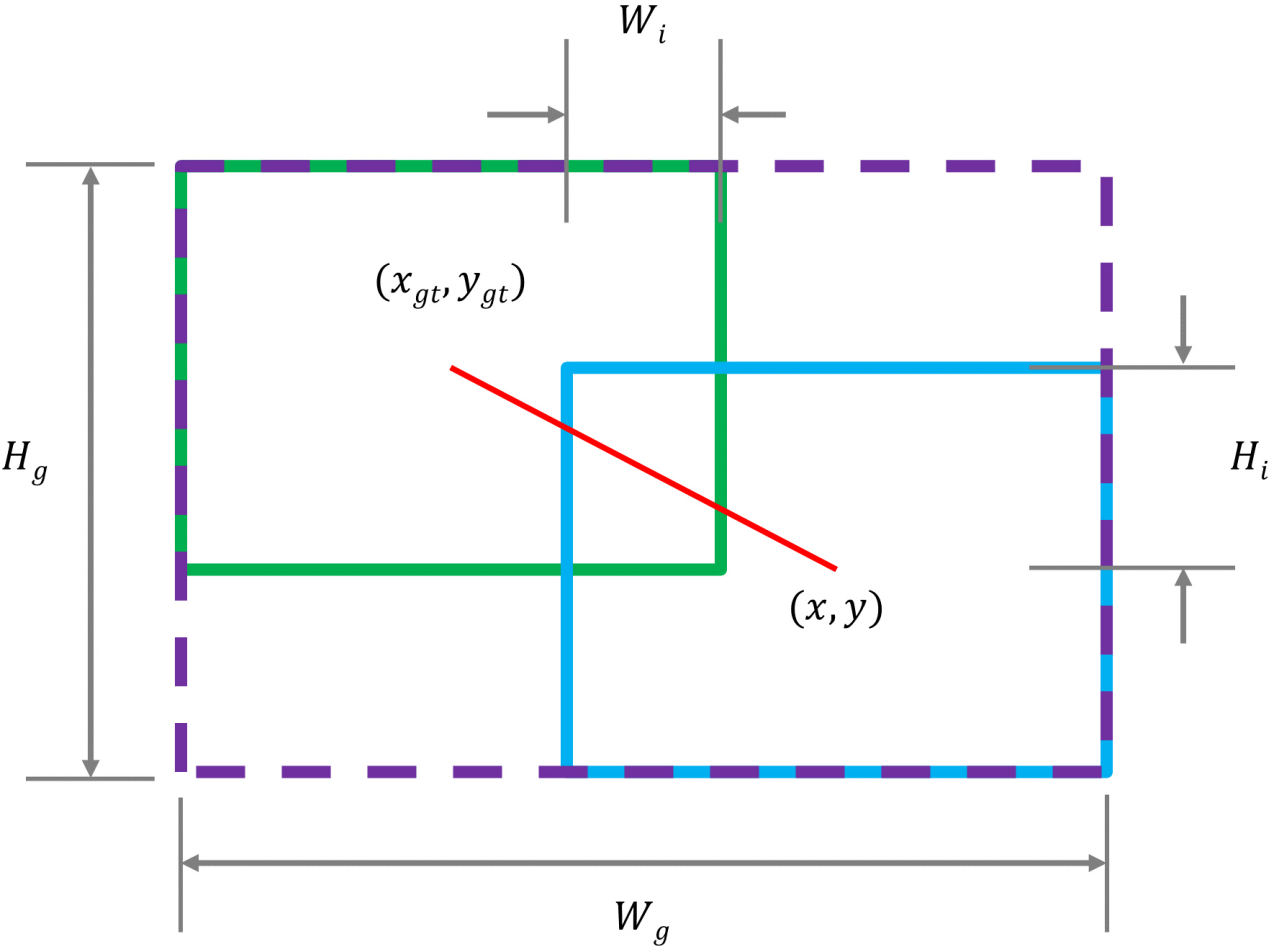
Parameter information when true and predicted bounding boxes are overlapped,the overlapped area can be expressed as $S_{u}=wh+w_{gt}h_{gt}-W_{i}H_{i}$ .


For SDTs detection using the YOLOv7 model, the CIoU is utilized to calculate the BBR loss of the target. **Figure 8** shows the parameter information when the true and predicted bounding boxes are overlapped, and Equation 5 is used to calculate the IoU loss. Equation 6 is constructed to represent the BBR loss, where the penalty term $R_{i}$ is used to measure the effect of geometric factors on the BBR loss. Zheng, Z et al. (Zheng et al., 2022) simultaneously considered three geometric elements, namely the intersection and concurrency ratio of the two bounding boxes, the distance from the centroid and the aspect ratio. They constructed both *L*_*CIoU*_ and *R*_*CIoU*_ (as illustrated in Equations 7, 8). The equations are shown as follows.

(5) $${ℒ}_{IoU}=1-IoU=1-\frac{W_{i}H_{i}}{S_{u}}$$

(6) $${ℒ}_{i}={ℒ}_{IoU}+{ℛ}_{i}$$

(7) $${ℒ}_{CIoU}={ℒ}_{IoU}+{ℛ}_{CIoU}$$

(8) $${ℛ}_{CIoU}=\frac{{\left(x-x_{gt}\right)}^{2}+{\left(y-y_{gt}\right)}^{2}}{W_{g}^{2}+H_{g}^{2}}+\alpha v$$

(9) $$\alpha =\frac{v}{{ℒ}_{IoU}+v},\;v=\frac{4}{\pi^{2}}{\left({\text{tan}}^{-1}\frac{w}{h}-{\text{tan}}^{-1}\frac{w_{gt}}{h_{gt}}\right)}^{2}$$

In Equation 9, *α* denotes the balance parameter and v  is used to measure the consistency of the aspect ratio. Labeling inaccuracies will inevitably occur when labeling a large amount of data, resulting in the appearance of some low-quality examples, and the computation of the localization loss will not be able to reduce the competitiveness of the low-quality examples if the geometric factors are taken into account, which affects the generalization performance of the model. To mitigate the impact of low-quality examples, this paper replaces the CIoU loss function in the YOLOv7 network with the WIoUv3 loss function (Tong et al., 2023). By using WIoUv3 , the interference caused by low-quality examples during model training is reduced, improving the overall performance of the model. The following equations show the calculation of the WIoUv1 loss based on attention (as illustrated in Equations 10, 11).


(10) $${ℒ}_{WIoUv1}={ℛ}_{WIoU}{ℒ}_{IoU}$$

(11) $${ℛ}_{WIoU}=\text{exp}\left(\frac{{\left(x-x_{gt}\right)}^{2}+{\left(y-y_{gt}\right)}^{2}}{{\left(W_{g}^{2}+H_{g}^{2}\right)}^{*}}\right)$$

Among them, ${ℛ}_{WIoU}$ can increase the model’s focus on common examples, and * denotes the separate separation of the length $W_{g}\;$ and width $H_{g}$ of the enclosing box, which serves to barely affect the convergence of the ${ℛ}_{WIoU}$ even if geometrical factors such as aspect ratios are not taken into account.

Based on the dynamic non-monotonic focusing mechanism, the degree of difference of the anchor box is denoted by β (as illustrated in Equations 12). The mechanism primarily focuses on prioritizing common examples, reducing the gradient gain allocated to high- and low-quality examples, and preventing larger gradients of low-quality examples from interfering with the BBR. The coefficient r  is set to construct the WIoUv3 loss function (as illustrated in Equations 13).


(12) $$\beta =\frac{{ℒ}_{IoU}^{*}}{\bar{{ℒ}_{IoU}}}\in \left[0,+\infty \right)$$

(13) $${ℒ}_{WIoUv3}=r{ℒ}_{WIoUv1},r=\frac{\beta }{\delta \alpha^{\beta -\delta }}$$

When β=δ r=1 β varies with ${ℒ}_{IoU}$ , allowing the gradient gain assignment criterion to adapt accordingly. This ensures that WIoUv3 can dynamically adjust the gradient gain assignment, giving greater attention to common examples in a timely manner.

Setting the momentum m bravely improves the focus on common examples early in model training (as illustrated in Equations 14), n  represents the total number of batches during training, and t  denotes the number of epochs at which the IoU loss approaches convergence.


(14) $$m=1-\sqrt[{tn}]{0.05}$$

By increasing the model’s focus on average-quality examples, the interference caused by low-quality examples to the BBR is reduced, resulting in a more rapid and smooth BBR convergence to enhance the model’s detection performance.

### 2.4 Experimental environment and training parameter

The experimental environment and parameter settings used to train the model during the experiment are shown in the **Table 2**.

**Table 2:** Experimental environment and training parameter.

Name	Specification	Name	Value
CPU	Intel(R) Xeon(R) Gold 6354	Optimizer	Adam
GPU	NVIDIA GeForce RTX 3090	Epochs	300
Operating System	Ubuntu 18.04	Learning Rate	0.001
Computing Platform	CUDA 11.1	Weight Decay	0.0005
Framework	Pytorch 1.8.2	Momentum	0.937
Language	Python 3.8	Batch Size	16

Model training from scratch will lead to slow convergence and poor results. In this paper, pretrained weights were used to accelerate the convergence of the model during training when experiments were conducted using the SDTs dataset. The initial learning rate is set to 0.001, the cosine fire reduction strategy is used to adjust the learning rate, the adaptive size of the image is set to 640*640, and 300 epochs of training are performed.

### 2.5 Evaluation metrics

The model detection performance was evaluated by comparing the magnitude of precision (P), recall (R), mean average precision (mAP) and frames per second (FPS) for detecting SDTs images before and after the model improvement, while ensuring that the experimental environments were the same. The precision represents the proportion of positive targets among all targets predicted by the model, and the recall represents the proportion of positive targets among all ground-truth targets predicted by the model (as illustrated in **Equations 15**, **16**).


(15) $$P=\frac{TP}{TP+FP}$$

(16) $$R=\frac{TP}{TP+FN}$$

According to the true target bounding boxes and predicted target bounding boxes, they are categorized into true positive cases, false positive cases, false negative cases, and true negative cases, and their corresponding numbers of detection boxes are denoted by TP, FP, FN, and TN, respectively. Neither precision nor recall metrics alone can show the detection capability of the model.

In order to comprehensively evaluate the detection performance of the model, the P-R curve is drawn with R as the vertical coordinate and P (the maximum P value is taken when R is the same) as the vertical coordinate, and the area surrounded by the curve and the coordinate axis is recorded as the average precision (AP) of single-category object detection. The mean Average Precision (mAP) represents the mean of AP of each category, which is calculated by Equations 17, 18:


(17) $$AP=\int_{0}^{1}PdR$$

(18) $$mAP=\frac{\sum_{i}^{N}AP_{i}}{N}$$

Where N is the number of target categories, in this paper, the detection target category is only SDTs, so AP = mAP in the following.

## 3 Results

### 3.1 Comparison of different attention mechanisms

To validate the effectiveness of the improved algorithm with the introduction of the attention mechanism, this paper employed the SimAM attention mechanism and compared it with another simple yet effective module, the Parameter-Free Average Attention Module (PfAAM)(Körber, 2022). We further compared these mechanisms to the Squeeze-and-Excitation Networks (SE) channel attention mechanism (Hu et al., 2018), the hybrid attention mechanism Convolutional Block Attention Module (CBAM) (Woo et al., 2018), and Coordinate Attention (CA) (Hou et al., 2021), which incorporates location information into channel attention. In order to ensure a rigorous and effective comparison, different attention mechanisms are added to the same network position while keeping the rest of the network structure unchanged. The experimental environment and model training parameters are kept consistent, and the weights are loaded for testing and comparison after training. The experimental results are presented in the **Table 3**.

**Table 3:** Comparison of detection results of different attention mechanisms.

Attention Mechanisms					Parameters (M)	*P*(%)	*R*(%)	*mAP* @0.5 (%)	*mAP* @0.5:0.95(%)	FPS
SE	CBAM	CA	PfAAM	SimAM						
					35.47	90.64	90.85	96.47	73.17	122
√					35.8	91.65	91.53	97.03	73.58	105
	√				35.89	91.53	89.63	96.36	72.62	79
		√			35.76	91.46	92.01	97.13	74.03	93
			√		35.47	91.64	92.73	97.29	74.08	109
				√	35.47	92.89	92.03	97.48	74.14	112

From **Table 3**, it can be observed that the CBAM_YOLOv7 model has the highest number of parameters compared to the YOLOv7 model, with an increase of 0.42M. It shows a slight improvement of 0.89% in precision, a decrease of 1.22% in recall, a decrease of 0.11% in mAP@0.5, and a significant reduction in detection speed by 43 FPS. The SE_YOLOv7 and CA_YOLOv7 model exhibits improved performance in all metrics except for a decrease in detection speed and an increase in the number of parameters. The SE_YOLOv7 model shows a 1.01% increase in precision value, a 0.68% increase in recall value, and a 0.56% increase in mAP@0.5 value compared to the YOLOv7 model. The CA_YOLOv7 model shows a 0.82% increase in precision value, a 1.16% increase in recall value, and a 0.66% increase in mAP@0.5 value compared to the YOLOv7 model. Without adding more parameters, PfAAM_YOLOv7 demonstrated improvements of 1% in precision value, 1.88% in recall value, and 0.82% in mAP@0.5 value compared to YOLOv7. The SimAM_YOLOv7 model demonstrates optimal performance in all metrics, enhancing precision by 2.25%, recall by 1.18%, and mAP@0.5 by 1.01%. Additionally, the SimAM_YOLOv7 model achieves the highest detection speed among the five models with attention mechanisms, reaching 112 FPS.

**Figure 9:**
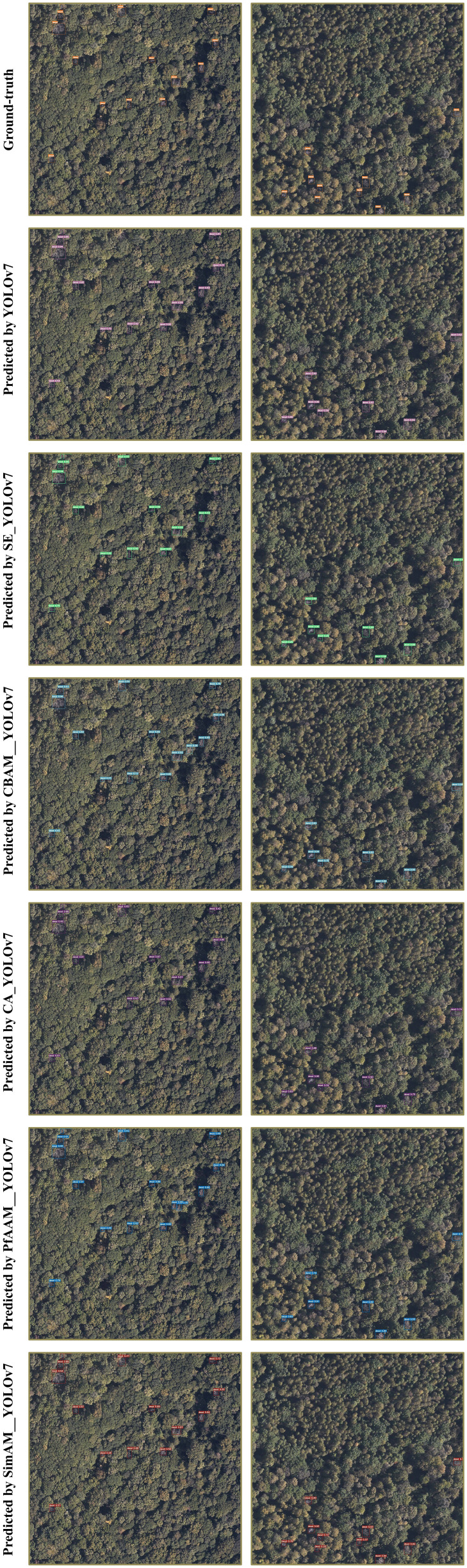
Ground-truth and prediction result.

Through the comparison and analysis of the experimental results, it can be concluded that the SimAM_YOLOv7 model exhibits better detection performance compared to the original YOLOv7 model, as well as the SE_YOLOv7, CBAM_YOLOv7, CA_YOLOv7 and PfAAM_YOLOv7 models, except for a slightly lower detection speed compared to the original YOLOv7 model. Furthermore, to further verify the impact of introducing different attention mechanisms on the model’s detection performance, the detection results are visualized and compared by loading images from the test set for each model. Some detection results are shown in **Figure 9**.

From the images in Group (A), it can be observed that for small target images, YOLOv7, SE_YOLOv7, CBAM_YOLOv7 and CA_YOLOv7 all exhibit instances of leakage detection, with one target missed detection. CBAM_YOLOv7 mistakenly identifies a tree with a similar color to the target as a dead standing tree, while PfAAM_YOLOv7 incorrectly identifies one dead tree as two separate instances. In Group (B), YOLOv7 misses two targets, and the visualization results indicate that even after the introduction of SE, CBAM and CA attention mechanisms, there are still instances of leakage when detecting small targets, with two targets missed detection, respectively. After the introduction of the PfAAM attention mechanism, three targets were missed. However, when using the SimAM_YOLOv7 model to detect SDTs, it successfully detects all ground-truth targets in the two test images. This suggests that it is capable of achieving comprehensive and accurate detection for images with complex backgrounds, similar colors and small targets.

### 3.2 Comparison of loss functions

**Figure 10:**
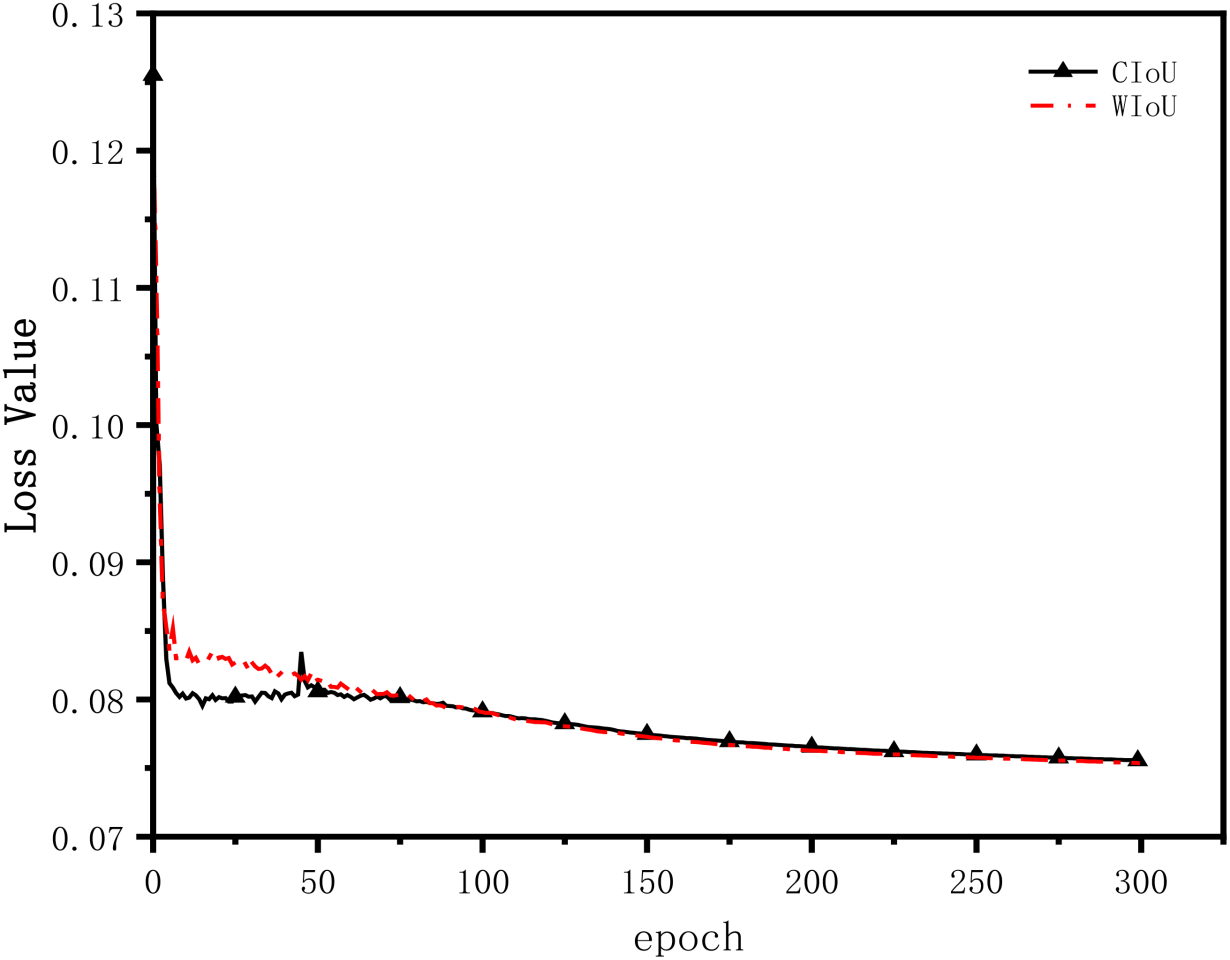
Loss function iteration comparison.

The impact of different BBR loss functions on model convergence is evaluated un-der identical experimental conditions and model training parameters. In this paper’s experiments, the CIoU and WIoU losses are introduced into the YOLOv7 model for comparison. The YOLOv7 model with the CIoU loss is denoted as YOLOv7-CIoU, while the YOLOv7 model with the improved loss function is referred to as YOLOv7-WIoU. The change curves of the two types of bounding box localization losses during the training process are depicted in **Figure 10**.

From the **Figure 10**, it is evident that both the CIoU loss and the WIoU loss reach convergence before 10 epochs during the training process. However, the WIoU loss converges faster and exhibits greater stability compared to the CIoU loss. Starting from the 87th epoch, the BBR loss of YOLOv7-WIoU becomes lower than that of YOLOv7-CIoU, and the discrepancy between the two loss values further amplifies in subsequent training. Eventually, at the end of training, the BBR loss values for YOLOv7-WIoU and YOLOv7-CIoU are 0.07537 and 0.07556, respectively, indicating a reduction of 0.00019 in the WIoU loss compared to the CIoU loss. In summary, this experiment validates the effectiveness of utilizing the WIoU loss and demonstrates its significance in enhancing the model’s performance.

### 3.3 Ablation experiments

The improvement algorithm proposed in this paper focuses on two key enhancements. To further evaluate the algorithm’s performance, we integrated SimAM into the backbone and neck of the YOLOv7 model for separate comparisons. Ablation experiments are conducted by loading the weights of the trained model with different improvement points into the network. The experiments are divided into six groups, controlling variables while ensuring that the experimental environment and training parameters remain unchanged. The resulting experimental outcomes are presented in the **Table 4**.

**Table 4:** Results of ablation experiments.

YOLOv7	SimAM			WIoU	*P*(%)	*R*(%)	*mAP* @0.5(%)	*mAP* @0.5:0.95(%)	FPS
	Backbone	Neck							
√					90.64	90.85	96.47	73.17	122
√	√				92.73	91.94	97.16	73.38	115
√		√			92.12	91.84	96.95	73.20	114
√	√	√			92.89	92.03	97.48	74.14	112
√				√	93.26	91.6	97.34	74.77	123
√	√	√		√	94.31	93.13	98.03	74.94	108

The experimental results of integrating SimAM into the YOLOv7 backbone and neck, respectively, indicate that both embedding methods enhance the model’s detection performance. The addition of SimAM to the backbone significantly enhanced the model’s detection accuracy. Specifically, the precision, recall and mAP@0.5 values increased by 0.61%, 0.1%, and 0.21% respectively, compared to the improvements observed in the neck. However, it is clear that embedding SimAM in both backbone and neck is more effective in improving the detection performance.

The SimAM_YOLOv7 model, integrating SimAM into both the backbone and neck of the YOLOv7 architecture, improves performance across all metrics compared to the original YOLOv7 model, except for a reduction in detection speed by 10FPS. It achieves this improvement by extracting feature information through calculating the corresponding weights of each neuron in the feature map. By replacing the CIoU loss with the WIoU loss in YOLOv7, the convergence speed of the model is improved, and all performance indexes show improvement compared to the original YOLOv7 model. Specifically, the precision, recall and mAP@0.5 values are enhanced by 2.62%, 0.75%, and 0.87% respectively, while the detection speed improves by 1 FPS.

**Figure 11:**
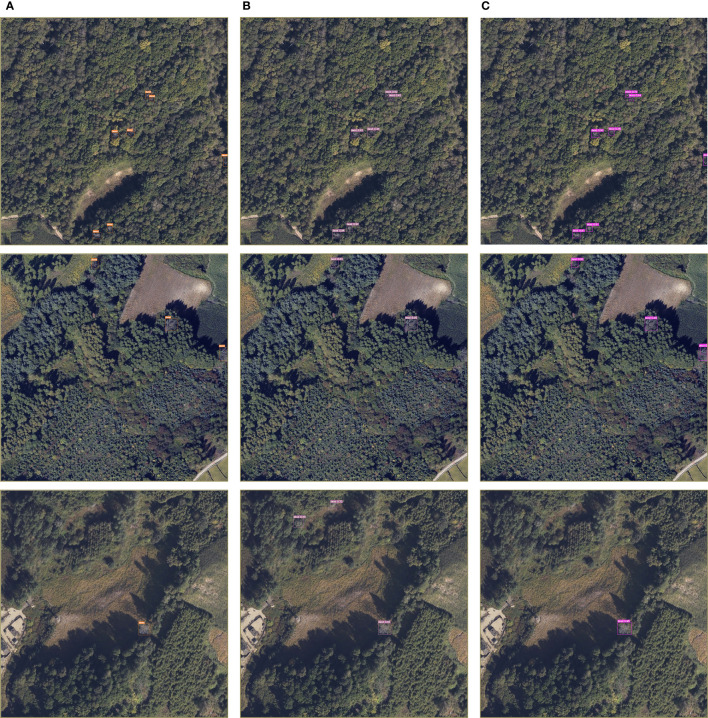
YOLOv7 model and improved model prediction results; **(A)** Ground-truth. **(B)** Predicted by YOLOv7. **(C)** Predicted by ours.

Furthermore, when both the SimAM parameter-free attention mechanism and the WIoU loss are introduced, the proposed algorithm demonstrates significantly superior performance compared to the other models. Compared to the original YOLOv7 model, the proposed algorithm shows improvements of 3.67% in precision value, 2.28% in recall value, and 1.56% in mAP@0.5 value, while the detection speed decreases by 14 FPS. These results effectively meet the real-time and accuracy requirements for SDTs detection. **Figure 11** presents some detection results from the YOLOv7 model and the proposed model in this paper on the SDTs dataset.

According to the **Figure 11**, when using the YOLOv7 model to detect dead trees, there are instances of leakage and misdetection. For example, in group (B) images, the targets on the right edge of the first and second images are not detected, and in the third picture, the model mistakenly detects similarly colored land and healthy standing trees as SDTs.

However, when comparing the results of the improved model proposed in this paper in group (C) images, it shows better detection performance for targets at the image edges and effectively improves the confidence level of detecting dead trees. The improved model is less affected by complex backgrounds and similar standing tree colors. The visualization results in **Figure 11** demonstrate that introducing the attentional mechanism and the WIoU loss in this paper without increasing model parameters enhances the overall performance of the model, despite a slight reduction in detection speed. The proposal of the improved model is of great significance for further research on SDTs detection and forest resource preservation.

### 3.4 Comparison experiments

**Figure 12:**
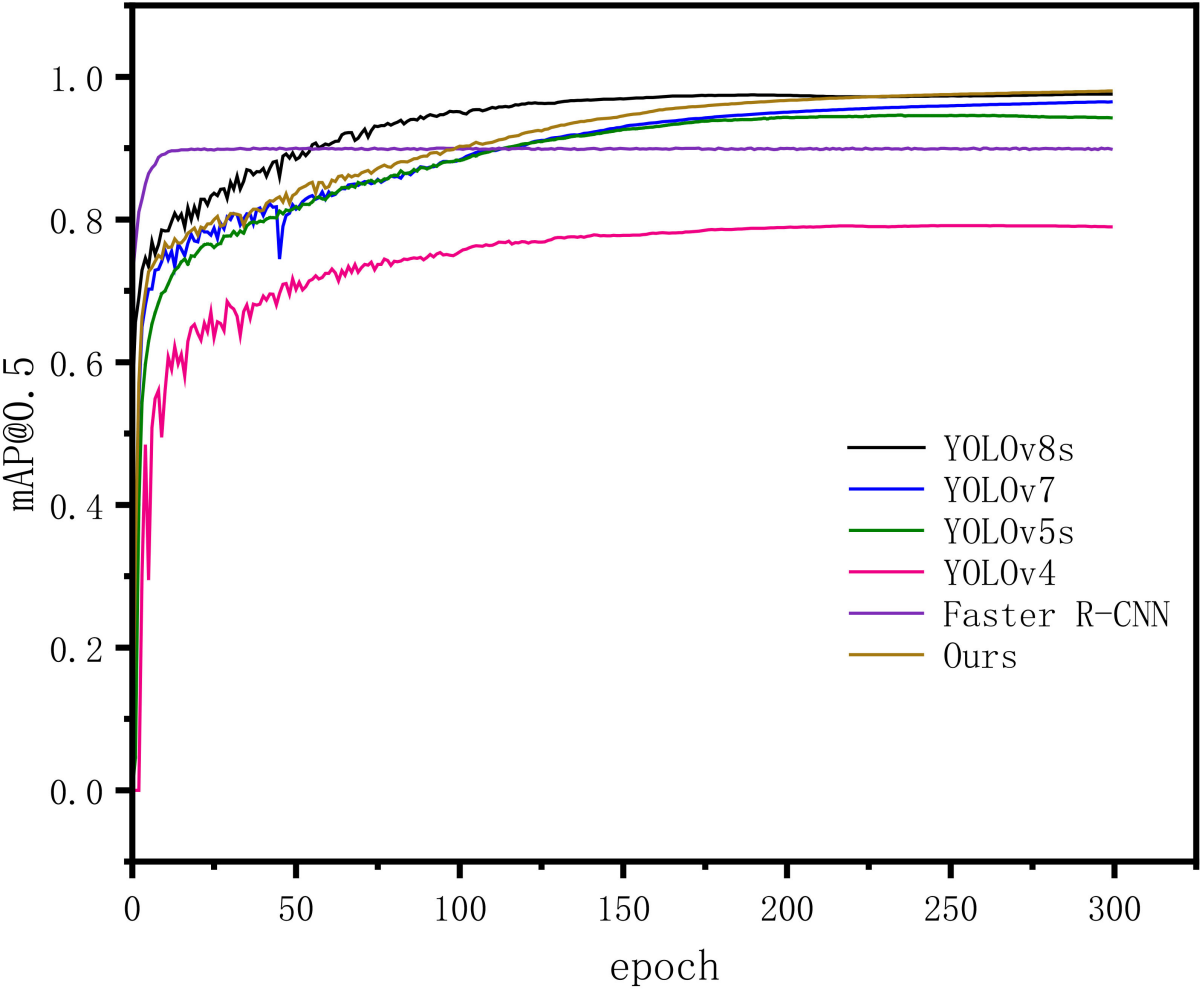
Different model training processes.

In order to substantiate the superiority of the proposed enhanced model, we conducted a comparative analysis with other commonly used algorithmic models under identical experimental conditions and dataset. The **Figure 12** illustrates the mAP@0.5-value change curve for each model during the training process, while **Table 5** presents the results of the comparative experiments.

**Table 5:** Results of comparison experiments.

Model	Backbone	*mAP* @0.5(%)	*mAP* @0.5:0.95(%)	FPS
YOLOv8s	Darknet53	97.58	74.62	127
YOLOv7	Darknet53	96.47	73.17	122
YOLOv5s	Darknet53	94.59	65.18	112
YOLOv4	Darknet53	79.15	49.06	50
Faster R-CNN	ResNet50_FPN	89.89	54.59	31
Ours	SimAM_ Darknet53	98.03	74.94	108

The figure illustrates that in this experiment, all models were trained for 300 epochs. It’s evident that throughout the training process, the detection accuracy of the models (typically denoted by mAP@0.5) steadily enhances, ultimately reaching a stable state. This signifies the convergence of the model training process. Faster R-CNN demonstrates the quickest increase in mAP@0.5 compared to other models and stabilizes first. The final mAP@0.5 values for each model align with the results presented in **Table 5**.

From the **Table 5**, it is evident that the proposed model in this paper achieves a higher mAP@0.5 value of 98.03% on the SDTs dataset, outperforming other main-stream models. Only YOLOv5s, YOLOv7 and YOLOv8s exhibit detection accuracies above 90%, which are lower by 3.44%, 1.56% and 0.45% respectively, compared to the improved model proposed in this paper. Among the other models, the Faster R-CNN model with Res-Net50+FPN as the backbone network demonstrates the highest mAP@0.5 value of 89.89%, while the detection mAP@0.5 value of the YOLOv4 models does not exceed 80%. These results verify the effectiveness and superiority of the improved model proposed in this paper in terms of dead trees detection accuracy.

In terms of model detection speed, specifically real-time detection performance, only four models, including the proposed model, achieve a speed higher than 100 FPS. The proposed model in this paper exhibits a detection speed of 108 FPS, which is slightly lower than YOLOv8s, YOLOv7 and YOLOv5s models by19, 14 and 4 FPS, respectively. However, it still holds a significant advantage over other models and fully satisfies the real-time demand for SDTs detection. Considering the detection mAP@0.5 and speed across various models, single-stage YOLO models, including YOLOv5 and subsequent versions, exhibit significant advantages over two-stage Faster R-CNN models. Our proposed improved model, in particular, surpasses other models in terms of detection performance. The potential reasons for the superior performance of our proposed model are twofold: Firstly, the SimAM attention mechanism significantly enhances the model’s ability to extract individual features of standing dead trees without introducing additional parameters. This reduction in interference from complex backgrounds and variations in the scale of dead trees during the detection process alleviates issues of both missed detections and false positives, thereby improving the accuracy of small target detection. Secondly, the replacement of the CIoU loss function with WIoU enhances the model’s robustness by focusing on bounding boxes of ordinary quality. This improvement accelerates the model’s convergence speed, further enhancing the accuracy of automatic detection of standing dead trees in high-resolution aerial remote sensing images.

### 3.5 Impact of different sized datasets on model performance

To verify the effectiveness of the data augmentation method employed in this study, 1928 images were taken as the basis, and the data volume was expanded by 2 times and 4 times, respectively. The three datasets were then used to train the im-proved YOLOv7 model. The experimental results are summarized in the **Table 6**.

**Table 6:** Comparison of different data volumes.

Number	*P*(%)	*R*(%)	*mAP* @0.5(%)	*mAP* @0.5:0.95(%)	FPS
1928	72.48	69.40	76.70	44.52	108.40
5784	85.46	86.15	92.47	64.86	108.28
9640	94.31	93.13	98.03	74.94	108.53

**Figure 13:**
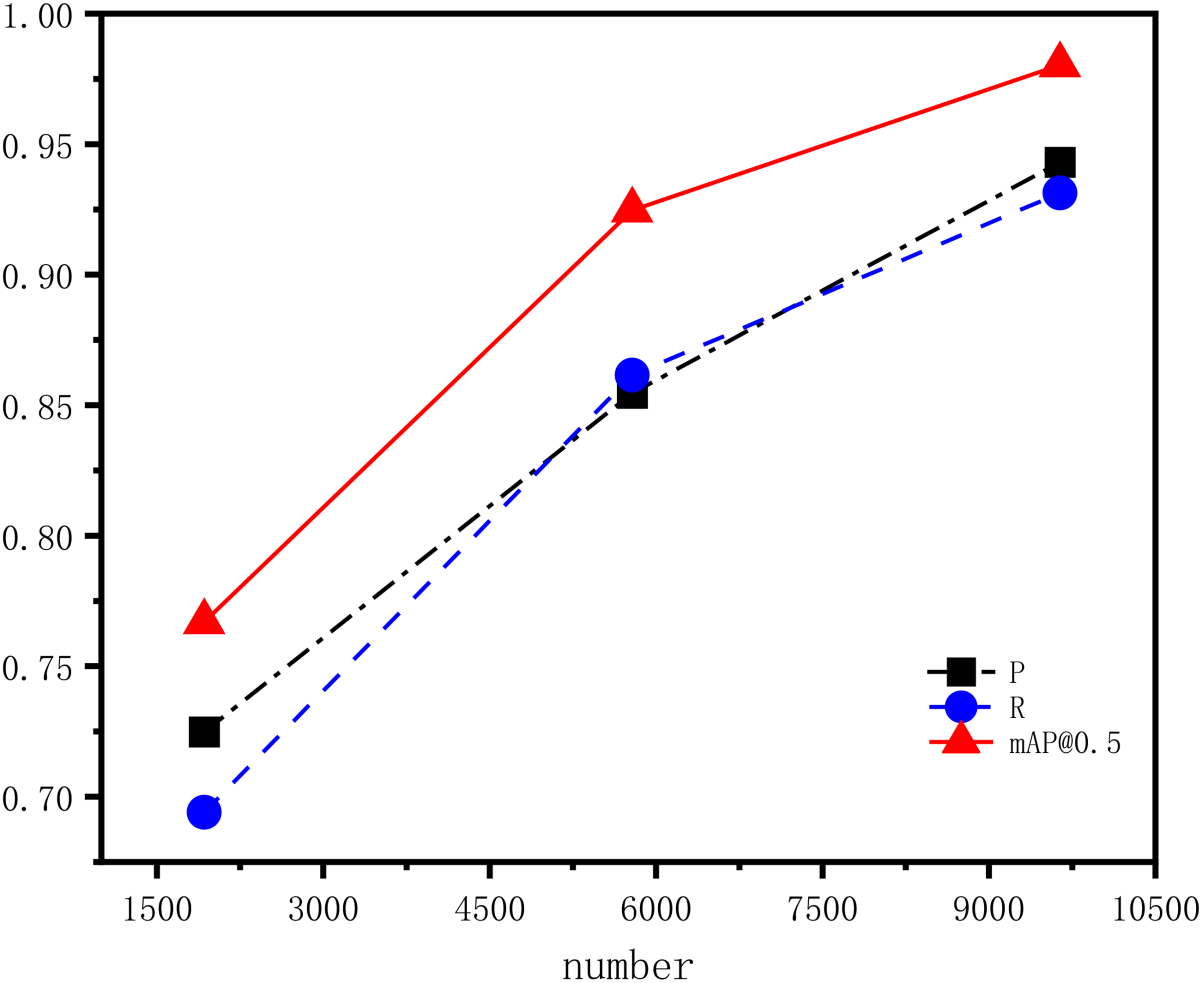
The detection results and metric trends for datasets of different sizes.

The **Table 6** shows that as the amount of data input into the model increases, the values of precision, recall, and mAP@0.5 gradually increase, but the growth rate slows down. When the dataset size reaches 9640 images, which was prepared for this study, the indicators reach their maximum values: precision at 94.31%, recall at 93.13%, and mAP@0.5 at 98.03%. The **Figure 13** illustrates the corresponding precision, recall, and mAP@0.5 metrics for the three datasets, along with their trends of change.

## 4 Discussion

Efficient and accurate automated identification of SDTs in forests is crucial for safeguarding forest resources and biodiversity. Conventional dead trees detection methods often encounter challenges such as difficulty, high expenses, and limited timeliness. To address these issues, this paper combines airborne remote sensing and deep learning techniques to achieve real-time and efficient automated identification of individual SDTs.

In this study, we utilized airborne remote sensing images with a ground resolution of 0.12 m, captured from an altitude of 1,000 m, as the data source. To meet the accuracy and real-time requirements, we proposed the improved YOLOv7 model for automated identification of dead trees. The model achieved precision, recall, mAP@0.5 and FPS values of 94.31%, 93.13%, 98.03%, 108 respectively. In similar studies of dead tree detection, Chiang et al. applied transfer learning to the Mask RCNN network for automated detection of SDTs in aerial images (Chiang et al., 2020). Li et al. pro-posed the LLAM-MDCNet method based on the MDCN network to detect clusters of dead trees in aerial images, aiming to reduce the interference from complex back-grounds and variable target scales by introducing the LIAM attention module (Li et al., 2022). However, their methods did not achieve the recognition of individual dead trees. To address the issue, Jiang et al. improved the Faster R-CNN algorithm based on the Swin-Transformer to enhance the learning of global information, enabling the recognition of individual SDTs in UAV images (Jiang et al., 2023). However, due to the two-stage nature of the Faster R-CNN algorithm, involving candidate region extraction, target classification and bounding box regression (BBR), the detection speed is slower, making it challenging to meet real-time requirements for SDTs detection (Ren et al., 2017). In an effort to improve the trade-off between accuracy and efficiency, Wang et al. proposed the LDS-YOLO method, which reduced the number of model parameters by enhancing the backbone network and introducing the SoftPool method into the SPP module. The accuracy of dead trees detection achieved with LDS-YOLO was reported as 89.11%, meeting the real-time requirements for automated dead trees detection (Wang et al., 2022). However, there is room for improvement in terms of the accuracy of detecting small targets. Compared to previous studies, the model proposed in this paper exhibits superior detection performance in both accuracy and speed when identifying small-target dead trees at the individual tree scale.After screening the dataset, it became evident that the sample size was insufficient. To enhance the model’s robustness and detection performance, data augmentation techniques such as random flipping, mirroring, and brightness adjustments were applied. Comparative experiments were conducted using the improved YOLOv7 model with varying data volumes. The results indicate that the mAP@0.5 value is positively correlated with the data volume, especially when the data volume is limited. The provided sample dataset in this paper reveals a dense distribution of trees in the Forestry Farm, with small and scattered canopies of dead trees. Challenges arise when recognizing dead trees, especially when shadows cast by tall trees obscure them, and in complex backgrounds where distinguishing dead trees becomes difficult. Visualizations in **Figures 9** and **11** indicate omissions and misdetections when using the YOLOv7 model, suggesting that the model has not fully learned the distinctive features of dead trees. This study addresses this issue by introducing SimAM and WIoU, which extract more detailed features from the limited dataset and improve the model’s focus on bounding boxes of average quality. Consequently, the detection accuracy of the model is significantly enhanced. Further-more, the improved YOLOv7 model exhibits faster detection speed compared to other mainstream models. This capability is crucial for rangers to monitor forest dynamics in real-time, effectively manage and protect forest resources.

This study has several limitations. Firstly, the improved model may face occlusion by healthy trees when recognizing dead trees, leading to misdetection and reducing the accuracy of the model. To address this issue, referring to Hell, M (Hell et al., 2022) and Wing, BM (Wing et al., 2015) for the detection of dead trees from LiDAR data, we can consider combining LiDAR data with optical remote sensing data. LiDAR data can provide information on tree height, shape, and point-cloud density, while optical remote sensing data can capture texture, color, and spectral features of the trees. By fusing the two types of features, we can achieve more accurate detection and localization of individual dead trees, thereby enhancing the accuracy of dead trees detection. Moreover, researchers have explored diseased and dead trees detection using multi-temporal multispectral images, ALS data and CIR images (Kaminska et al., 2018; Wu et al., 2023), which could serve as valuable references for our future research.

Another limitation of this study is the insufficient consideration of external environmental factors that may interfere with the experiment. Variations in lighting conditions and resolution can cause changes in the color and texture of SDTs, leading to detection interference. To mitigate the impact of these factors, we can use multiple image views or collect multi-temporal remote sensing images under different environmental conditions. This approach will provide more comprehensive tree contrast information and improve the robustness of the detection model.

To sum up, our proposed automatic SDTs detection model holds promising applications in forest protection and disaster prevention. In the future, we will explore and develop more real-time and efficient dead trees detection methods by incorporating multiple data sources, thereby further enhancing the accuracy and applicability of the model.

## 5 Conclusions

This study demonstrates the potential of deep learning algorithms in detecting SDTs from airborne remote sensing images. To overcome the limitations of traditional manual inventory methods, we propose an automatic detection model based on an improved YOLOv7 for efficient identification and localization of dead trees in remote sensing images. The model, built upon YOLOv7, addresses challenges posed by dense canopies and complex forest backgrounds by embedding the SimAM attention mechanism module in the backbone and neck. Compared to embedding the other four attention mechanisms in YOLOv7, the SimAM_YOLOv7 model has a smaller number of parameters and achieves higher detection accuracy. Additionally, The WIoU loss function is employed instead of the CIoU loss to enhance the model’s focus on ordinary labeled samples, improving convergence speed and detection accuracy. The experimental results reveal that the improved model achieves precision, recall, and mAP@0.5 values of 94.31%, 93.13%, and 98.03%, respectively, representing a 3.67%, 2.28%, and 1.56% improvement over the original YOLOv7 model. Furthermore, the model outperforms other mainstream models in terms of the combined performance of detection accuracy and speed. The proposed model holds practical applications in forestry management, offering a convenient solution for forest resource and biodiversity conservation.

## Data availability statement

The raw data supporting the conclusions of this article will be made available by the authors, without undue reservation.

## Author contributions

SW: Conceptualization, Formal analysis, Methodology, Project administration, Writing – review & editing. HZ: Conceptualization, Formal analysis, Methodology, Validation, Writing – original draft, Writing – review & editing. ZX: Conceptualization, Project administration, Writing – review & editing. HS: Conceptualization, Formal analysis, Project administration, Writing – review & editing.

## References

[B1] Bernal A. A. Kane J. M. Knapp E. E. Zald H. S. J. (2023). Tree resistance to drought and bark beetle-associated mortality following thinning and prescribed fire treatments. For. Ecol. Manage. 530, 120758. doi: 10.1016/j.foreco.2022.120758

[B2] Butler R. Schlaepfer R. (2004). Spruce snag quantification by coupling colour infrared aerial photos and a GIS. For. Ecol. Manage. 195, 325–339. doi: 10.1016/j.foreco.2004.02.042

[B3] Celik T. (2009). Unsupervised change detection in satellite images using principal component analysis and k-means clustering. IEEE Geosci. Remote Sens. Lett. 6, 772–776. doi: 10.1109/LGRS.2009.2025059

[B4] Chai B. Nie X. Gao H. Jia J. Qiao Q. (2023). Remote sensing images background noise processing method for ship objects in instance segmentation. J. Indian Soc. Remote Sens. 51, 647–659. doi: 10.1007/s12524-022-01631-7

[B5] Chen G. Hay G. J. Castilla G. St-Onge B. Powers R. (2011). A multiscale geographic object-based image analysis to estimate lidar-measured forest canopy height using Quickbird imagery. Int. J. Geogr. Inf. Sci. 25, 877–893. doi: 10.1080/13658816.2010.496729

[B6] Chiang C.-Y. Barnes C. Angelov P. Jiang R. (2020). Deep learning-based automated forest health diagnosis from aerial images. IEEE Access 8, 144064–144076. doi: 10.1109/ACCESS.2020.3012417

[B7] Coops N. C. Gillanders S. N. Wulder M. A. Gergel S. E. Nelson T. Goodwin N. R. (2010). Assessing changes in forest fragmentation following infestation using time series Landsat imagery. For. Ecol. Manage. 259, 2355–2365. doi: 10.1016/j.foreco.2010.03.008

[B8] Eklundh L. Johansson T. Solberg S. (2009). Mapping insect defoliation in Scots pine with MODIS time-series data. Remote Sens. Environ. 113, 1566–1573. doi: 10.1016/j.rse.2009.03.008

[B9] Farias G. Fabregas E. Dormido-Canto S. Vega J. Vergara S. Dormido Bencomo S.. (2018). Applying deep learning for improving image classification in nuclear fusion devices. IEEE Access 6, 72345–72356. doi: 10.1109/ACCESS.2018.2881832

[B10] Girshick R. (2015). ”Fast R-CNN,” in 2015 IEEE International Conference on Computer Vision (ICCV), Santiago, Chile, 2015, 1440–1448. doi: 10.48550/arXiv.1504.08083

[B11] Han Z. Hu W. Peng S. Lin H. Zhang J. Zhou J.. (2022). Detection of standing dead trees after pine wilt disease outbreak with airborne remote sensing imagery by multi-scale spatial attention deep learning and gaussian kernel approach. Remote Sens. 14, 3075. doi: 10.3390/rs14133075

[B12] Hell M. Brandmeier M. Briechle S. Krzystek P. (2022). Classification of tree species and standing dead trees with lidar point clouds using two deep neural networks: pointCNN and 3DmFV-net. PFG-J. Photogramm. Remote Sens. Geoinf. Sci. 90, 103–121. doi: 10.1007/s41064-022-00200-4

[B13] Hicke J. A. Logan J. (2009). Mapping whitebark pine mortality caused by a mountain pine beetle outbreak with high spatial resolution satellite imagery. Int. J. Remote Sens. 30, 4427–4441. doi: 10.1080/01431160802566439

[B14] Hou Q. Zhou D. Feng J. (2021). “Coordinate attention for efficient mobile network design,” in 2021 IEEE/CVF Conference on Computer Vision and Pattern Recognition (CVPR), Nashville, TN, USA, 2021, 13708–13717. doi: 10.1109/CVPR46437.2021.01350

[B15] Hu J. Shen L. Sun G. (2018). Squeeze-and-excitation networks. Available at: https://openaccess.thecvf.com/content_cvpr_2018/html/Hu_Squeeze-and-Excitation_Networks_CVPR_2018_paper.html (Accessed July 14, 2023).

[B16] Jiang X. Wu Z. Han S. Yan H. Zhou B. Li J. (2023). A multi-scale approach to detecting standing dead trees in UAV RGB images based on improved faster R-CNN. PloS One 18, e0281084. doi: 10.1371/journal.pone.0281084 36827399 PMC9956600

[B17] Kaminska A. Lisiewicz M. Sterenczak K. Kraszewski B. Sadkowski R. (2018). Species-related single dead tree detection using multi-temporal ALS data and CIR imagery. Remote Sens. Environ. 219, 31–43. doi: 10.1016/j.rse.2018.10.005

[B18] Kim Y.-J. Verghese P. (2012). The selectivity of task-dependent attention varies with surrounding context. J. Neurosci. 32, 12180–12191. doi: 10.1523/JNEUROSCI.5992-11.2012 22933800 PMC6621517

[B19] Körber N. (2022). Parameter-free average attention improves convolutional neural network performance (Almost) free of charge. doi: 10.48550/arXiv.2210.07828

[B20] Lee C. K. F. Song G. Muller-Landau H. C. Wu S. Wright S. J. Cushman K. C.. (2023). Cost-effective and accurate monitoring of flowering across multiple tropical tree species over two years with a time series of high-resolution drone imagery and deep learning. ISPRS-J. Photogramm. Remote Sens. 201, 92–103. doi: 10.1016/j.isprsjprs.2023.05.022

[B21] Lee S. Park S. Baek G. Kim H. Lee C.-W. (2019). Detection of damaged pine tree by the pine wilt disease using UAV image. Korean J. Remote Sens. 35, 359–373. doi: 10.7780/KJRS.2019.35.3.2

[B22] Lei T. Li L. Lv Z. Zhu M. Du X. Nandi A. K. (2021). Multi-modality and multi-scale attention fusion network for land cover classification from VHR remote sensing images. Remote Sens. 13, 3771. doi: 10.3390/rs13183771

[B23] Li Y. Li J. Meng P. (2023). Attention-YOLOV4: a real-time and high-accurate traffic sign detection algorithm. Multimed. Tools Appl. 82, 7567–7582. doi: 10.1007/s11042-022-13251-x

[B24] Li Z. Yang R. Cai W. Xue Y. Hu Y. Li L. (2022). LLAM-MDCNet for detecting remote sensing images of dead tree clusters. Remote Sens. 14, 3684. doi: 10.3390/rs14153684

[B25] Luo Y. Huang H. Roques A. (2023). Early monitoring of forest wood-boring pests with remote sensing. Annu. Rev. Entomol. 68, 277–298. doi: 10.1146/annurev-ento-120220-125410 36198398

[B26] Manning A. D. Fischer J. Lindenmayer D. B. (2006). Scattered trees are keystone structures - Implications for conservation. Biol. Conserv. 132, 311–321. doi: 10.1016/j.biocon.2006.04.023

[B27] Maxwell A. E. Warner T. A. Fang F. (2018). Implementation of machine-learning classification in remote sensing: an applied review. Int. J. Remote Sens. 39, 2784–2817. doi: 10.1080/01431161.2018.1433343

[B28] Meng J. Li S. Wang W. Liu Q. Xie S. Ma W. (2016). Mapping forest health using spectral and textural information extracted from SPOT-5 satellite images. Remote Sens. 8, 719. doi: 10.3390/rs8090719

[B29] Miltiadou M. Agapiou A. Gonzalez Aracil S. Hadjimitsis D. G. (2020). Detecting dead standing eucalypt trees from voxelised full-waveform lidar using multi-scale 3D-windows for tackling height and size variations. Forests 11, 161. doi: 10.3390/f11020161

[B30] Nadrowski K. Wirth C. Scherer-Lorenzen M. (2010). Is forest diversity driving ecosystem function and service? Curr. Opin. Environ. Sustain. 2, 75–79. doi: 10.1016/j.cosust.2010.02.003

[B31] Naga Srinivasu P. Krishna T. B. Ahmed S. Almusallam N. Khaled Alarfaj F. Allheeib N. (2023). Variational autoencoders-basedSelf-learning model for tumor identification and impact analysis from 2-D MRI images. J. Healthcare Eng. 2023, 1–17. doi: 10.1155/2023/1566123 PMC9873460 36704578

[B32] Niu Z. Zhong G. Yu H. (2021). A review on the attention mechanism of deep learning. Neurocomputing 452, 48–62. doi: 10.1016/j.neucom.2021.03.091

[B33] Redmon J. Divvala S. Girshick R. Farhadi A. (2016). “You only look once: unified, real-time object detection,” in 2016 IEEE Conference on Computer Vision and Pattern Recognition (CVPR), Las Vegas, NV, USA, 2016, 779–788. doi: 10.48550/arXiv.1506.02640

[B34] Ren S. He K. Girshick R. Sun J. (2017). Faster R-CNN: towards real-time object detection with region proposal networks. IEEE Trans. Pattern Anal. Mach. Intell. 39, 1137–1149. doi: 10.1109/TPAMI.2016.2577031 27295650

[B35] Rezatofighi H. Tsoi N. Gwak J. Sadeghian A. Reid I. Savarese S. (2019). “Generalized intersection over union: A metric and a loss for bounding box regression,” in 2019 IEEE/CVF Conference on Computer Vision and Pattern Recognition (CVPR), Long Beach, CA, USA, 2019, 658–666. doi: 10.1109/CVPR.2019.00075

[B36] Sirisha U. Praveen S. P. Srinivasu P. N. Barsocchi P. Bhoi A. K. (2023). Statistical analysis of design aspects of various YOLO-based deep learning models for object detection. Int. J. Comput. Intell. Syst. 16, 126. doi: 10.1007/s44196-023-00302-w

[B37] Srivastava P. Shukla A. Bansal A. (2021). A comprehensive review on soil classification using deep learning and computer vision techniques. Multimed. Tools Appl. 80, 14887–14914. doi: 10.1007/s11042-021-10544-5

[B38] Tong Z. Chen Y. Xu Z. Yu R. (2023). Wise-ioU: bounding box regression loss with dynamic focusing mechanism. doi: 10.48550/arXiv.2301.10051

[B39] Voulodimos A. Doulamis N. Doulamis A. Protopapadakis E. (2018). Deep learning for computer vision: A brief review. Comput. Intell. Neurosci. 2018, 1–13. doi: 10.1155/2018/7068349 PMC5816885 29487619

[B40] Wang C.-Y. Bochkovskiy A. Liao H.-Y. M. (2022). “YOLOv7: Trainable bag-of-freebies sets new state-of-the-art for real-time object detectors,” in 2023 IEEE/CVF Conference on Computer Vision and Pattern Recognition (CVPR), Vancouver, BC, Canada, 2023, 7464–7475. doi: 10.48550/arXiv.2207.02696

[B41] Wang J. Deng J. Yan W. Zheng Y. (2023). Habitat suitability of pine wilt disease in northeast China under climate change scenario. Forests 14, 1687. doi: 10.3390/f14081687

[B42] Wang Y. Zhang W. Gao R. Jin Z. Wang X. (2021). Recent advances in the application of deep learning methods to forestry. Wood Sci. Technol. 55, 1171–1202. doi: 10.1007/s00226-021-01309-2

[B43] Wang X. Zhao Q. Jiang P. Zheng Y. Yuan L. Yuan P. (2022). LDS-YOLO: A lightweight small object detection method for dead trees from shelter forest. Comput. Electron. Agric. 198, 107035. doi: 10.1016/j.compag.2022.107035

[B44] Wang H. Zhao Y. Pu R. Zhang Z. (2015). Mapping robinia pseudoacacia forest health conditions by using combined spectral, spatial, and textural information extracted from IKONOS imagery and random forest classifier. Remote Sens. 7, 9020–9044. doi: 10.3390/rs70709020

[B45] Windrim L. Carnegie A. J. Webster M. Bryson M. (2020). Tree detection and health monitoring in multispectral aerial imagery and photogrammetric pointclouds using machine learning. IEEE J. Sel. Top. Appl. Earth Observ. Remote Sens. 13, 2554–2572. doi: 10.1109/JSTARS.2020.2995391

[B46] Wing B. M. Ritchie M. W. Boston K. Cohen W. B. Olsen M. J. (2015). Individual snag detection using neighborhood attribute filtered airborne lidar data. Remote Sens. Environ. 163, 165–179. doi: 10.1016/j.rse.2015.03.013

[B47] Woo S. Park J. Lee J.-Y. Kweon I. S. (2018). “CBAM: convolutional block attention module,” in 2018 European Conference on Computer Vision (ECCV), Munich, Germany, 2018, 3–19. doi: 10.1007/978-3-030-01234-2_1

[B48] Wu D. Yu L. Yu R. Zhou Q. Li J. Zhang X.. (2023). Detection of the monitoring window for pine wilt disease using multi-temporal UAV-based multispectral imagery and machine learning algorithms. Remote Sens. 15, 444. doi: 10.3390/rs15020444

[B49] Yang L. Zhang R.-Y. Li L. Xie X. (2021). “SimAM: A simple, parameter-free attention module for convolutional neural networks,” in Proceedings of the 38th International Conference on Machine Learning Proceedings of Machine Learning (ICML), Vienna, Austria, 2021, 11863–11874. Available at: https://proceedings.mlr.press/v139/yang21o.html.

[B50] Zhang S. Song F. Lei T. Jiang P. Liu G. (2022). MKLM: a multiknowledge learning module for object detection in remote sensing images. Int. J. Remote Sens. 43, 2244–2267. doi: 10.1080/01431161.2022.2061316

[B51] Zheng Z. Wang P. Ren D. Liu W. Ye R. Hu Q.. (2022). Enhancing geometric factors in model learning and inference for object detection and instance segmentation. IEEE T. Cybern. 52, 8574–8586. doi: 10.1109/TCYB.2021.3095305 34437079

[B52] Zheng J. Yuan S. Wu W. Li W. Yu L. Fu H.. (2023). Surveying coconut trees using high-resolution satellite imagery in remote atolls of the Pacific Ocean. Coord. Chem. Rev. 481, 113485. doi: 10.1016/j.rse.2023.113485

